# New York Letter

**Published:** 1892-11

**Authors:** 


### Correspondence.

### NEW YORK LETTER.

NEW YORK, Oct. 1892.

Though the excitement caused by the recent, near approach, and indeed the entrance of cholera into our country has passed away, and there is but little fear of its immediate return, it has started a train of argument in professional minds, as well as the minds of all thinking men, that should not be allowed to abate until some practical result is obtained. This train of thought is, how shall we prevent the entrance of infectious diseases into our land from foreign countries? This question does not interest those situated along the seacoast alone, but is of the deepest import to those living inland, for once let such a disease as cholera, typhus, or yellow fever get a foothold in one of the large seaport towns, quarantine as you will, you cannot prevent its spread from city to city and from State to State.

Much has been said *pro* and *con*, upon the administration of quarantine in New York during its late threatened invasion by the dreaded disease. It must be remembered that the true state of affairs in Europe had been concealed until the pest laden ships were almost in our port. The sudden emergency, therefore, arose when there was no time to prepare for it, and even when the disease was in our harbor, the majority were skeptical of its identity. Until Fire Island was obtained the ship cabins were the most comfortable quarters that could be had for the quarantined. Had the health officer asked the legislature for $200,000 to provide a quarantine station with last winter, does any one suppose it would have been granted? Or if it had been granted what a howl about deal, political corruption fund, etc., would have arisen. In this emergency it was only by the moral courage and intrepid bravery of a humanitarian governor that the means were put into the health officer's hands to abate the hardships of the quarantined. Though the laws enforced by Dr. Jenkins, at times, seemed harsh or even cruel, it was the best that could be done under the circumstances, and, while we do not wish to be thought jesuitical, we think the end justified the means, and the question now is, how can we obtain as good results with less harsh measures.

The lively interest that is being taken in this question was evidenced at a recent meeting of the New York County Medical Association, where papers were read by some of the most prominent professional men of our country.

Dr. Chas. A. Leale read a paper on the subject of "Cholera, its propagation and treatment," in which he said that cholera is endemic in Hindoostan, and from there it travels to other parts of the world and after a time disappears as suddenly as it came. Why it should remain in Hindoostan and disappear from other countries so suddenly and completely, can no more be answered than why after a forest fire an entirely different species of tree will spring up; but why it spreads so rapidly in that country is explained by the fact that when an inhabitant is stricken with the dreaded disease he is placed in a little, poorly ventilated room and his friends gather around him simply to pray, without making the least effort at medical treatment, and without pretending to protect themselves or the patient from the vomit or discharge from the bowels. The disease is thus carried from house to house until it becomes epidemic. The germs of the disease are carried by transportation to other countries, through clothing and articles of merchandise, where they get into the water and food of the cities. Dr. Sternberg, in a recent paper read before the New York County Medical Society, said that there is little danger of the disease being carried through the mails, as after being dried the germ loses its vitality to a large extent. However, Dr. Dunham, at the meeting above mentioned, presented cultures of the cholera spirillum made from dry rags.

The cholera spirillum does not thrive in the gastric juice, but let it reach a stomach in which the acidity is diminished from any reason, and it immediately begins to grow, and passing into the intestines where the alkaline fluids favor its development, it multiplies rapidly, and the ptomains get into the blood and cause the nervous symptoms observed. The vomiting and diarrhœa caused by the irritation of the germs, so reduces the serum in the blood that the heart's action is impeded.

If a case of cholera is seen in the early stage, it is often easily overcome, but unfortunately, it passes so rapidly from one stage to another that we do not frequently see it early. During the stage of collapse, the patient should be placed in a recumbent position and surrounded by dry heat, the heart stimulated, and the fluid in the blood increased by flushing the alimentary canal, through the rectum, with a weak saline solution at about 98 degs. F. For the cramps, alternately compressing and relaxing the limbs by grasping them with the hands is, according to Dr. Leale, the best method of treatment. He has seen patients recover after having been in a state of collapse for four hours.

The physicians in Hamburg have found the best results, in extreme cases of collapse, to be obtained by the intra-venous injection of warm saline solutions.

Dr. E. K. Dunham gave a microscopical and stereoscopical demonstration of the cholera spirillum and the methods of cultivating it, the specimens and cultures being made from the cases which occurred in this city.

Drs. Godfrey and Gihon, of the U. S. Marine Service, spoke of the advantages of quarantine and the methods of conducting it.

Dr. Godfrey said that the object of quarantine was to destroy germs or to prevent their coming to a country. The latter method simply means to stop intercourse while the former would only retard commerce. The former method would be decidedly the best if we only knew how to destroy the germs of all infectious diseases. But we do not know this. Dr. Sternberg, probably the greatest authority in this country, says we do not know the germs of yellow fever and other fevers, and therefore, we do not know what will destroy them. Who knows but that the germs of yellow fever will thrive in bichloride of mercury and grow fat in sulphur fumes?

The ideal quarantine station is one equipped with the best methods for disinfection, with a hospital for the sick and a house for the well, with a laboratory equipped for the scientific study of germs, so that we will not have to depend on the "guess work of clinical symptoms" for diagnosis, and manned by scientific workers not appointed by political poll, and subject to removal at every election, but by competitive examination, to hold office during life or good behavior. Such a quarantine office, supported by the United States government, would serve to prevent, or at least to diminish, the annual visitations of yellow fever in the Southern States, and exclude the dangers of cholera, typhus fever and other infectious diseases entering our country. But unfortunately, quarantine is looked upon by many, in a purely mercenary manner, and they raise the question, "which will cost the most, the cessation of immigration and commerce for a time, or an epidemic?" and as those in power think that the danger is but slight for them, they decide that the epidemic is the least expensive, until one of these little insignificant germs finds it's way into his household, and then, too late, he changes his mind. But says Dr. Godfrey, "I wish you to understand that I consider the life of an individual of more value than all commerce."

Dr. Smith read a paper on choleraic diarrhœa in infants, in which he stated that during a cholera epidemic, it is often quite impossible to say, without a microscopical examination, whether or not a given case is cholera infantum or Asiatic cholera. He attaches but little importance to the rice-water stools in such cases.

At a recent meeting of the Section in Public Health of the New York Academy of Medicine, the cholera question was minutely discussed. In a paper by Dr. Joseph H. Raymond, the efficacy of sulphur dioxide as a disinfectant against the cholera spirillum, was discussed. He presented statistics from the experiments of Drs. H. M. Biggs, L. H. Thoinot and M. Masselin, which showed that S O is an efficient disinfectant against the cholera spirillum. It should not be used, however, to the exclusion of bichloride and other antiseptics, but should follow their application. After an infected room has been cleansed with a 1-500 solution of corrosive sublimate, all the openings being carefully closed, it should be fumigated for several hours with SO, when it will be impossible to make a culture of the cholera germs from articles or scrapings from the room.

At the annual meeting of the American Orthopedic Association, Dr. H. Hodgen, of St. Louis, reported a case of adduction of the foot, following fracture of the neck of the femur. The case illustrated, in a marked manner, the difficulty of diagnosis in some cases, between fracture of the neck and dislocation of the head of the femur. It was treated by extension and a double-inclined plane.

Dr. H. A. Wilson, of Philadelphia, stated that the changes in the structure of bones, often described in club-foot, are, to a large extent, due to postponement of appropriate treatment. This may be accounted for in either of two ways. First, where the process of ossification progresses while the tarsal bones are partially dislocated, they become permanently deformed to suit the abnormal position of the foot; secondly, where recourse has been had to mechanical force to stretch the shortened tendons, the bones, whether deformed or not, yield to pressure, and thereby become deformed. He formulated the following principles:

1. Full, perfect correction should be obtained during the first month.

2. When correction is possible, without recourse to rigid restraining apparatus, tenotomy should be avoided.

3. Tenotomy, syndesmotomy, or cutting of the fascia, should always be resorted to within the first month, when perfect correction cannot otherwise be obtained without undue force.

4. Tenotomy should never be resorted to in infancy, without being followed by developmental and restraining treatment.

5. It is misapplied mechanics to force club-feet into rigid restraining shoes and doing so in the first three months of infancy, will produce bone deformity.

6. All apparatus employed in infancy, should facilitate free motion in the proper direction, and encourage the development and correlation of muscular force.

7. All methods which employ undue force in correcting or restraining club-feet, should be refrained from until the child has reached the age when bones are completely ossified. The same period should be reached before resorting to operations upon the bones.

Dr. J. E. Moore, of Minneapolis, reported six cases of excision of the knee joint, in which the incision above the patella was used, and the patella removed. Drainage tubes were not recommended. The bones were united by four steel wire nails. The plaster of Paris dressing extended from the toes to the body, and was not changed for one month.

At a recent meeting of the New York Pathological Society, Dr. J. S. Ely demonstrated, under a microscope, the amœba dysenteria in ulcers of the colon, and in an abscess of the liver. The demonstration was particularly interesting as it is the first time these amœba have been demonstrated in the tissues, though the whole subject has been carefully worked up and described by Drs. Councilman and LaFleur, of Johns Hopkins University. The speaker said, that on a previous occasion he had found the amœba present in the stools of a patient. The specimen had been stained with the ordinary stain of hæmatoxyline and eosin.

The study of the life history of this germ, it's viability, what medicaments will destroy it, and whether or not it will cause dysentery when placed in a healthy alimentary canal, is an interesting field for investigation. T. D. T.

---

REMEDY FOR STERILITY—Dr. Gritian, of Lyons, knowing the intimate connection existing between the uterus and the larynx, always told us in his lectures: "If ever you meet a woman complaining of infecundity, tell her to sing at the top of her voice during the copulative act, or the actus conjug; it is the best method of promoting conception." I gave this advice on two occasions to friends, and it proved a success, although one of them had a pair of twins. She told me, after that experience she kept her mouth shut.—*Medical Times.*

